# Investigation of inner ear drug delivery with a cochlear catheter in piglets as a representative model for human cochlear pharmacokinetics

**DOI:** 10.3389/fphar.2023.1062379

**Published:** 2023-03-09

**Authors:** Erdem Yildiz, Anselm J. Gadenstaetter, Matthias Gerlitz, Lukas D. Landegger, Rudolfs Liepins, Michael Nieratschker, Rudolf Glueckert, Hinrich Staecker, Clemens Honeder, Christoph Arnoldner

**Affiliations:** ^1^ Christian Doppler Laboratory for Inner Ear Research, Department of Otorhinolaryngology, Vienna General Hospital, Medical University of Vienna, Vienna, Austria; ^2^ Department of Otorhinolaryngology, Head and Neck Surgery, Vienna General Hospital, Medical University of Vienna, Vienna, Austria; ^3^ Department of Otorhinolaryngology, Medical University of Innsbruck, Innsbruck, Austria; ^4^ Department of Otolaryngology, Head and Neck Surgery, University of Kansas School of Medicine, Kansas, KS, United States

**Keywords:** drug delivery, large animal model, pharmacokinetics, SNHL = sensorineural hearing loss, inner ear catheter, pig, cochlear catheter, drug distribution

## Abstract

Hearing impairment is the most common sensory disorder in humans, and yet hardly any medications are licensed for the treatment of inner ear pathologies. Intricate pharmacokinetic examinations to better understand drug distribution within this complex organ could facilitate the development of novel therapeutics. For such translational research projects, animal models are indispensable, but differences in inner ear dimensions and other anatomical features complicate the transfer of experimental results to the clinic. The gap between rodents and humans may be bridged using larger animal models such as non-human primates. However, their use is challenging and impeded by administrative, regulatory, and financial hurdles. Other large animal models with more human-like inner ear dimensions are scarce. In this study, we analyzed the inner ears of piglets as a potential representative model for the human inner ear and established a surgical approach for intracochlear drug application and subsequent apical sampling. Further, controlled intracochlear delivery of fluorescein isothiocyanate-dextran (FITC-d) was carried out after the insertion of a novel, clinically applicable CE-marked cochlear catheter through the round window membrane. Two, six, and 24 hours after a single injection with this device, the intracochlear FITC-d distribution was determined in sequential perilymph samples. The fluorometrically assessed concentrations two hours after injection were compared to the FITC-d content in control groups, which either had been injected with a simple needle puncture through the round window membrane or the cochlear catheter in combination with a stapes vent hole. Our findings demonstrate not only significantly increased apical FITC-d concentrations when using the cochlear catheter but also higher total concentrations in all perilymph samples. Additionally, the concentration decreased after six and 24 hours and showed a more homogenous distribution compared to shorter observation times.

## Graphical Abstract

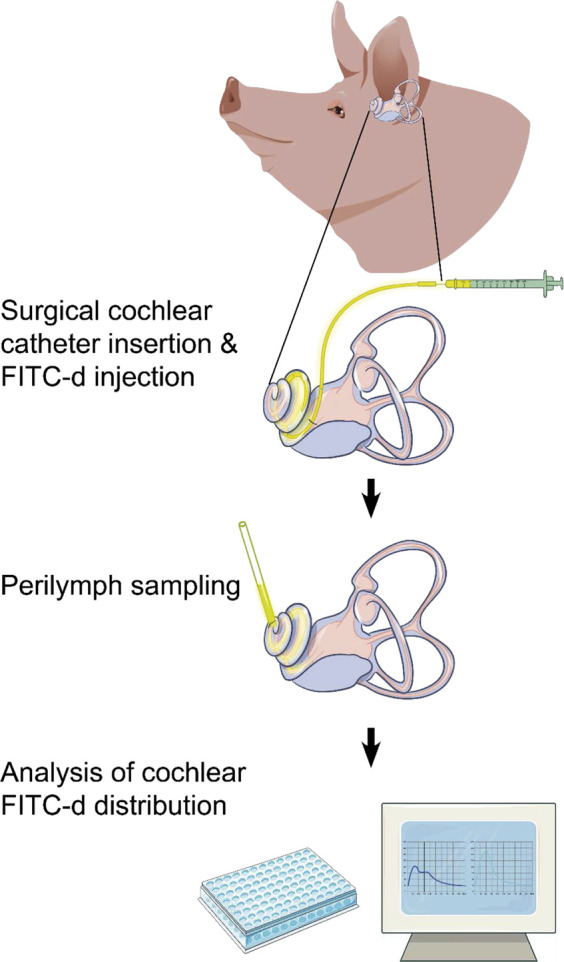

## 1 Introduction

**FIGURE 1:**
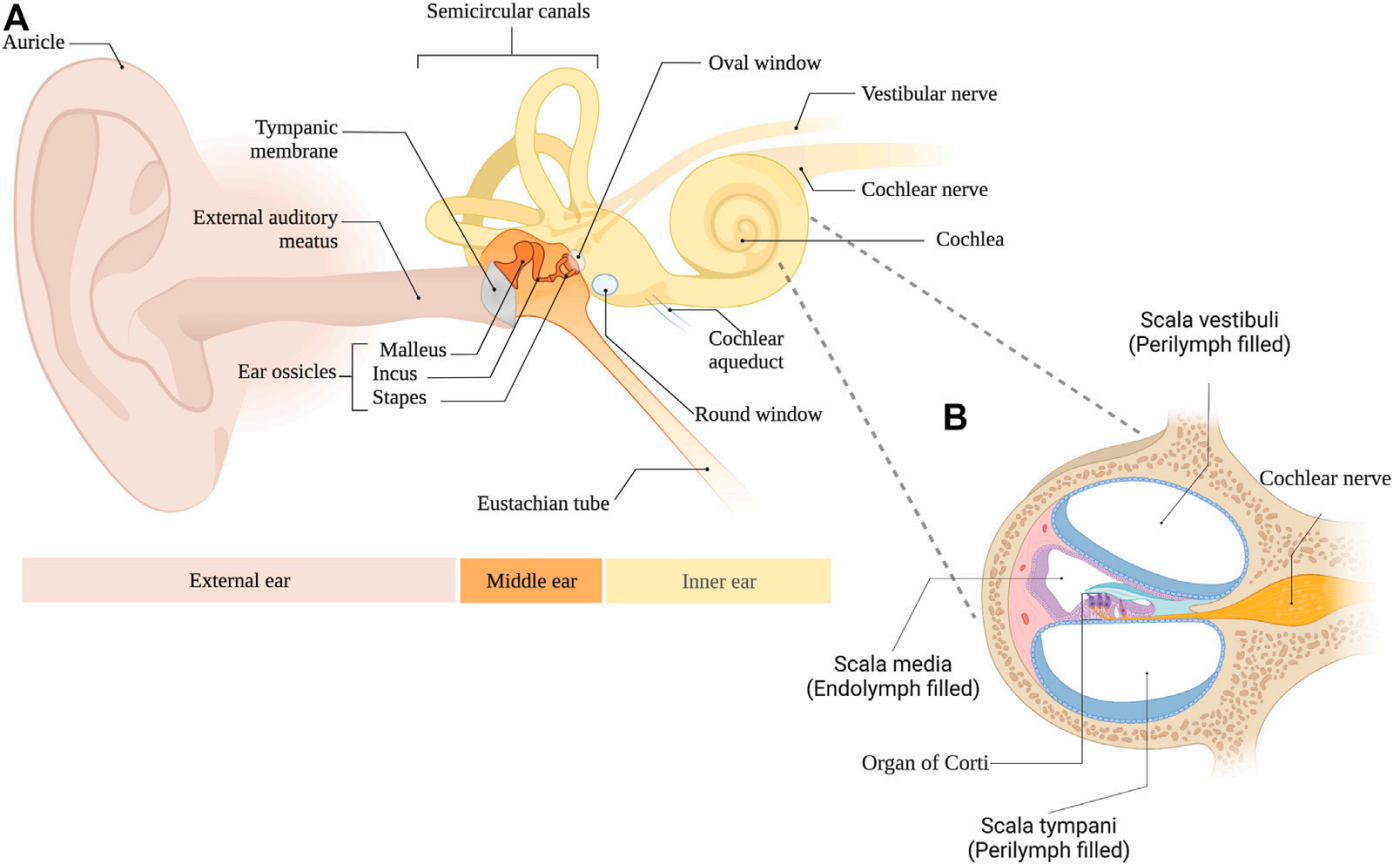
**(A)** Anatomical overview of the external, middle and inner ear. **(B)** Cross section of the cochlea.

Hearing loss is a major global health concern with more than 1.5 billion people affected and over 430 million of these suffering from disabling hypoacusis (WHO, 2022). Currently, no drugs are approved to treat the most common form of this condition, namely sensorineural hearing loss. As illustrated in Figure 1A, the hearing system can be divided into the external, middle, and inner ear as well as the central auditory pathway. Acoustic stimuli pass the ear canal and arrive at the tympanic membrane. In mammals, the vibrations of the tympanic membrane are further forwarded *via* three ossicles to the oval window, representing the connection to the fluid-filled inner ear. Due to this ossicular transmission, reflections at the surface of the cochlea are minimized and sound energy is efficiently delivered. The intricate process is further aided by the second membrane-covered opening of the inner ear, namely, the round window. This structure allows the displacement of perilymph (PL), which fills two cochlear compartments known as scala vestibuli and scala tympani (Figure 1B). In the inner ear, a traveling wave is generated, which, depending on the frequency of the acoustic stimulus, exhibits its maximum at different parts of the snail-shaped hearing organ. High frequencies are represented at the basal parts of the cochlea, i.e., close to the round window, while low frequencies result in maximal amplitudes of the traveling wave in the apical parts of the inner ear. This phenomenon, also known as tonotopy, is further enhanced by the three rows of outer hair cells, which act as amplifiers and fine-tune the signal. Finally, the adjacent inner hair cells depolarize and release glutamate at the ribbon synapse. This leads to the stimulation of the respective spiral ganglion neuron and to the generation of an action potential that is then further transferred to the cochlear nucleus, where central processing of the hearing impression begins. Even though many pathophysiological processes resulting in sensorineural hearing loss have been described, most of them involve the sensory cells, whose cell bodies are surrounded by PL while the surface of the so-called organ of Corti is bathed in endolymph.

The complex inner ear pharmacokinetics play an integral part in preventing a straightforward approach to developing novel therapeutics. The so-called blood-labyrinth barrier blocks an efficient allocation of systemically applied drugs into the perilymphatic and endolymphatic fluid spaces to a large extent (Salt and Plontke, 2018). Hence, the local delivery of compounds to avoid this barrier and directly apply substances to the middle or inner ear is of utmost clinical relevance for future therapeutic endeavors. Key features for local pharmacotherapy include the two membranous ports to the inner ear, the round window membrane (RWM) and the annular ligament of the oval window attached to the stapes footplate, permeability through multiple internal fluid compartments, and putative routes to the CSF across the cochlear aqueduct (CA). Currently, local drug delivery is usually achieved by the trans-/intratympanic injection of liquid drug carriers into the middle ear, from where the active compound passively enters the inner ear by diffusion. However, the efficacy of this process is limited by the rapid loss of fluids through the Eustachian tube, an open duct that connects the middle ear with the pharynx.

Only a small number of topically delivered medications with effects on the inner ear are widely used in routine clinical practice. These include aminoglycosides for the treatment of Meniere’s disease (Basura et al., 2020) and the therapeutic use of corticosteroids, e.g., in idiopathic sudden sensorineural hearing loss (Chandrasekhar et al., 2019; Dahm et al., 2021; Plontke et al., 2022). In the scope of local drug delivery to the inner ear, various medications such as antibiotics, steroids, antioxidants, biologicals (Roemer 2017), and promising gene or stem cell therapy alternatives are explored today (Staecker and Rodgers, 2013; Warnecke et al., 2020). Over the last decades, various hurdles have been overcome regarding intracochlear administration of such compounds, but further challenges need to be resolved (Liu and Yang, 2022). Surgical intervention is inevitable for intrascalar drug delivery *via* direct injection or an application in combination with the use of devices such as cochlear implants with bioactive electrodes (Hendricks et al., 2008; Ayoob and Borenstein, 2015; Plontke et al., 2017). Potential entry points into the inner ear include puncture of the round window or oval window or creating a separate opening (so-called cochleostomy). In contrast, a middle ear (intratympanic) drug injection is less invasive. Still, high doses must be applied due to Eustachian tube clearance and the fact that the membranous cochlear structures are not permeable to certain substances. Diffusion is low even for compounds that can penetrate the membranes (Chin and Diaz, 2019) and permeability changes with inflammatory responses. The CE-marked cochlear catheter (CC; MED-EL, Innsbruck, Austria) provides an alternative solution for the single injection of solvents in patients with inner ear pathologies. This medical device was recently tested in human patients and was proofed as a safe drug delivery method (Prenzler et al., 2018; Prenzler et al., 2020). Despite the clinical applicability of the catheter, its advantages compared to simple needle puncture through the RWM have not been evaluated *in vivo*.

The apical sampling methodology described in several previous publications (Mynatt et al., 2006; Salt et al., 2006; Manrique-Huarte et al., 2021) is a well-established standard for sequential inner ear fluid collection. This technique helps gauge concentration gradients along the cochlear compartments and can be used after intracochlear drug delivery (Mynatt et al., 2006). Despite the fact that cochlear fluid dynamics might be slightly altered after the perforation of the most apically oriented bone, this methodology provides significant advantages compared to PL collection through the RWM. It allows for sequential sampling of small fractions of PL that are thought to represent different parts of the inner ear and it limits the potential dilution of such samples by cerebrospinal fluid (CSF), the liquid surrounding the central nervous system. CSF is believed to enter the inner ear through the CA, connecting the subarachnoid space with the basal turn of the cochlea. Hence, the collection of PL in close proximity of the CA, e.g., through the RWM, appears to be more prone to dilution than when samples are obtained from other locations.

The neutral polysaccharide fluorescein isothiocyanate dextran (FITC-d) is a widely used compound that serves as an ideal drug distribution marker in the perilymph fluid space. Permeability of FITC-d conjugates with various molecular sizes (low and high molecular weights) can be tested to assess drug delivery through the blood-brain-barrier (Sharma et al., 2019). In guinea pigs, intracochlear FITC-d administration revealed that the oscillating exchange of PL and CSF *via* the CA plays a dominant factor in homeostasis (Salt et al., 2015). Considering the significant variance of cochlear fluid spaces between different species (Thorne et al., 1999), this and the longer and narrower CA in humans impedes the translation of findings from rodents to humans (Burton et al., 2019). To overcome this problem, large animal models, such as non-human primates (Balkany et al., 1980), cats (Snyder et al., 1991), sheep (Kaufmann et al., 2020) or minipigs (Yi et al., 2014) have been used in inner ear research. Most recently, the use of the porcine model appears to increase due to a variety of advantages compared to the other animals (Yi et al., 2016; Liu et al., 2021; Yildiz et al., 2022).

Therefore, FITC-d was injected after insertion of a CC through the RWM in a newly established large animal model with inner ear dimensions similar to humans. The main objectives of our study were to evaluate short-term molecular distribution profiles in piglet inner ears after single FITC-d administration *via* CC, and to compare this technique to a control group with simple needle puncture through the RWM under similar conditions. Together with the proposed novel porcine animal model, we strive to present findings with translational relevance for clinical therapies.

## 2 Materials and methods

### 2.1 Anesthesia and perioperative care

All piglets were mixed strains of domestic pigs (*Sus scrofa domesticus*) and animals of either sex were used for the experiments. The weight of the animals ranged between 8.35 and 20 kg (12.81 ± 2.64 kg, mean ± standard deviation, SD). A mix of ketamine (10 mg/kg body weight, BW), medetomidine (100 μg/kg BW), and midazolam (37.5 μg/kg BW) was injected intramuscularly behind the right ear. Each sedated animal was brought into the operating room in a calm environment, and a venous catheter was inserted into the right auricular vein. After intubation, anesthesia was maintained with 4% sevoflurane inhalation, and a constant body temperature was preserved between 38.3°C and 39.7°C with a bair hugger™ (3M, Saint Paul, Minnesota, USA). The intraoperative pain medication consisted of 10 μg/kg BW fentanyl i.v. and was adapted during surgical intervention. Perioperative antibiotic treatment was carried out with tulathromycin (2 mg/kg BW i.m.). Preventive antiemetic treatment consisted of the intravenous application of droperidol (50 μg/kg BW). All animals with 24 h of observation time between FITC-d injection and PL sampling received postoperative analgetic treatment with three piritramide injections (0.15 mg/kg BW i.m.) in equal intervals on the day of the surgical intervention. Additionally, metamizole (30 mg/kg BW i.v.) and 6 µg fentanyl patches were administered for postoperative pain prevention.

### 2.2 Objective auditory measurements

Before surgery, the left outer ear canal was cleared from extensive cerumen. All audiometric tests were performed with a mobile audiometry device (Intelligent Hearing Systems, Miami, Florida, USA). One subcutaneous needle electrode (–) was placed retroauricularly, and two additional needle electrodes (+) were placed on the vertex. Another needle electrode was placed subcutaneously on the snout and served as the ground electrode. All acoustic stimuli were presented in decreasing steps of 10 dB starting from 120 dB sound pressure level (SPL) to determine threshold levels. For click-evoked auditory brainstem responses (ABRs), the stimulus was presented with a duration of 0.1 ms, a rate of 19.3/sec, alternating polarity and the recordings were averaged following 512 sweeps. All click-ABRs were recorded prior to surgery to ensure functional hearing. To record electrocochleography (ECochG) signals, a gold wire electrode (PromStim^®^, MED-EL, Innsbruck, Austria) was placed on the RWM before and after FITC-d injection and fixed on the outer ear canal with tissue glue (Histoacryl^®^, B. Braun, Melsungen, Germany). In detail, acoustic compound action potential (CAP) thresholds of a click stimulus were determined. 4 kHz cochlear microphonic (CM) recordings were obtained after acoustic stimulation with each condensation and rarefaction polarity and subsequent separation of the ECochG signal. The 20 kHz tone burst CAP thresholds were determined after the application of the same method described above. CM recordings with 20 kHz auditory stimuli were not feasible due to the limited bandwidth of the measurement setup. The stimulus rate for both CAP and CM recordings was 35.1/sec with 400 sweeps. All recordings were analyzed after visualization with a custom-made code in MATLAB version R2022a (MathWorks, Inc., Massachusetts, USA), which was uploaded to an online repository (Liepins et al., 2022) on the GitHub website (San Francisco, California, USA).

### 2.3 Experimental setup

An endaural surgical approach for inner ear drug delivery, PL sampling, and CSF collection was established in the porcine cadaver model prior to *in vivo* experimentation. The applied compound consisting of 2.5 mmol/l FITC-d (4,000 g/l, Sigma Aldrich, St. Louis, Missouri, USA) was prepared in Minimal Essential Medium (MEM, no glutamine, no phenol red, Fisher Scientific, Waltham, Massachusetts, USA) under sterile conditions. The prepared solution was injected intraoperatively through the porcine RWM, either with a novel CE-marked CC (MED-EL, Innsbruck, Austria) or with a 23-gauge needle cannula (BD Vacutainer, Becton Dickinson, Franklin Lakes, New Jersey, USA), which were both connected to a micropump (UMP3T-1, World Precision Instruments LLC, Sarasota, Florida, USA) for controlled delivery. The CC has an outflow in the catheter tip, where it measures approximately 0.3 mm width. It contains an array of 20 mm length that has black markings at the measurements 5, 10, and 15 mm of electrode array, and the total device length is 12.6 cm. A small septum at the end of the catheter allows the attachment of a needle tip for injection of drugs. A volume of 40 µl FITC-d solution, which should theoretically result in the complete filling of the scala tympani of pigs (Table 1), was applied within 10 min (4 μl/min). Each group consisted of 5 animals, resulting in a total of 25. PL sampling was performed 2, 6, or 24 h after initial FITC-d injection *via* CC (abbreviated as *2h CC*, *6h CC*, and *24h CC,* respectively). Control groups were formed of animals that had been injected with FITC-d *via* simple needle puncture through the RWM (*2h RWM*) or the combination of a CC insertion through the RWM with a stapes vent hole (venting group, *2h Vent CC*). The venting group served as a comparative group to the conventional CC insertion due to potentially improved scalar FITC-d distribution after the outflow of excessive PL post injection. For the RWM injection, a 23-gauge needle cannula (BD Vacutainer, Becton Dickinson) was inserted through the RWM until the membrane covered the cannula bevel and no backflow of FITC-d was visible. The precise CC insertion depth of 12.5 mm was measured on the marked dots relative to the RWM. Prior to the injection of FITC-d into the inner ear, the round window was carefully sealed with subcutaneous tissue from the ear canal that had been prepared in advance during the course of the surgery. A meticulous closure of the round window prevented backflow of injected FITC-d. During the course of the 10-minute-long injection time, neither the catheter nor the surrounding tissue was manipulated in order to prevent any potential structural or sensory damage within the cochlea. After finishing the injection, the catheter was slowly removed from the inner ear, the round window was sealed with additional tissue and by the application of tissue glue. Once the observation time had passed (2, 6 or 24 h), the apical bone was visualized using a surgical microscope (PROvido, Leica Microsystems, Wetzlar, Germany) and meticulously thinned using an otologic drill (1.5 mm diamond drill, Stryker, Austria). Subsequently, tissue glue was applied, a silicone cup was formed, the apex was opened *via* microneedle puncture and PL was collected with glass microcapillaries.

**TABLE 1:** Volumetric measurements of cochlear compartments.

Cochlear compartment	Individual volume measurements (mm^3^)					Mean ± SD (mm^3^)
	Ear 1	Ear 2	Ear 3	Ear 4	Ear 5	
Scala tympani	35.63	28.02	25.61	33.38	27.93	30.11 ± 3.76
Scala media	10.52	6.82	7.94	8.59	6.75	8.12 ± 1.38
Scala vestibuli	26.85	23.71	19.33	19.71	18.23	21.57 ± 3.23
Cochlear aqueduct	1.30	2.68	1.08	1.20	3.14	1.88 ± 0.86
Semicircular canals	25.95	21.36	25.00	20.58	21.46	22.87 ± 2.17
Total inner ear volume	100.25	82.59	78.96	83.46	77.51	84.55 ± 8.15

Representative individual and collective volume measurements of piglet inner ears after PL sampling and following segmentation of micro-CT scans.

After sequential PL sampling (15 × 2 µl), all samples were stored at −20°C before concentration levels were determined with fluorometric measurements in a multiplate reader (Tecan Spark^®^, Tecan Trading, Maennersdorf, Switzerland) using a black 384-well plate with a lid (Thermo Fisher Scientific Inc., Waltham, Massachusetts, USA) with 25x dilution of PL or CSF in distilled water. A triplicate control fluorescent dilution series was prepared with the same FITC-d compound as injected and was used to determine an individual calibration curve for each well plate measurement. Throughout the experiments, care was taken to avoid extensive photobleaching.

### 2.4 Micro-CT imaging and cochlear segmentation

After PL sampling and subsequent euthanasia with pentobarbital (300 mg/kg BW), five piglet inner ears were treated with 4% formaldehyde (prepared freshly from paraformaldehyde) and consecutively postfixed in an osmium tetroxide solution (1%) overnight. The samples were sequentially dehydrated, fixed in a vial with surrounding gauze, and scanned using an Xradia MicroXCT-400 (Carl Zeiss X-ray Microscopy, Pleasanton, California, USA) with an isotropic voxel resolution of 8.3 μm at the VetCore Facility (University of Veterinary Medicine Vienna). Specimens were imaged over a 360° sample rotation using a 45 kVp/177 µA X-ray spectrum. Projection images were recorded with an exposure time of 10 seconds (camera binning = 1) and an angular increment of 0.225° between projections. All scans were imported into the software package AMIRA^®^ 6.0.1 (FEI Visualization Sciences Group, Mérignac Cédex, France). Each cochlear compartment was segmented manually in multiple planes for optimal volume definition. After the surface smoothing, the software calculated the volumetric data for each segmented cochlear compartment (scala tympani, scala media, scala vestibuli, semicircular canals, and CA) using the *Surface Area Volume* tool. Finally, a volume rendering (*Volren* tool) was performed to better visualize the location of each perfusion hole in the apical bone.

### 2.5 Statistical analysis

GraphPad Prism version 9.4.1 (GraphPad Software, San Diego, California, USA) statistical software was applied for data analysis in this study. The averaged FITC-d concentrations were reported in mean values and shaded error areas indicate the standard error of the mean (SEM) if not described otherwise in the figure legends. The 95% confidence interval was shown with the abbreviation CI. Inferential statistical analysis of hearing thresholds was performed using the paired t-test. Shapiro-Wilk test confirmed normal distribution between sampling groups in FITC-d concentration values and hearing thresholds. Multifactorial ANOVA analysis with *post hoc* Tukey was performed for comparison of individual FITC-d concentration values between each sampling group and mean concentration values of cochlear compartments defined as *apical* (sample number 1–3), *middle* (sampling number 5–8), and *basal* (sampling number 10–14). All described *p*-values are provided as adjusted *p*-values and were considered statistically significant when lower than 0.05.

## 3 Results

Click-ABR thresholds before surgery were successfully assessed in all animals, and recorded data served to identify the threshold for unimpaired cochlear function. The experimental paradigm consisting of the surgical approach with CC insertion (12.5 mm depth), controlled FITC-d application, and repeated objective hearing measurements was reproducible in all animals. Consecutive PL sampling was performed from the most apical part of the cochlea, which was visible with the surgical exposure obtained *via* the endaural approach. Collective PL (15 × 2 µl) sampling time ranged between 25 and 90 min (43 ± 16 min, mean ± SD). We did not detect any FITC-d in CSF samples collected *via* syringe punction into the cisterna magna (cerebellomedullar cistern).

### 3.1 Surgical approach

**FIGURE 2:**
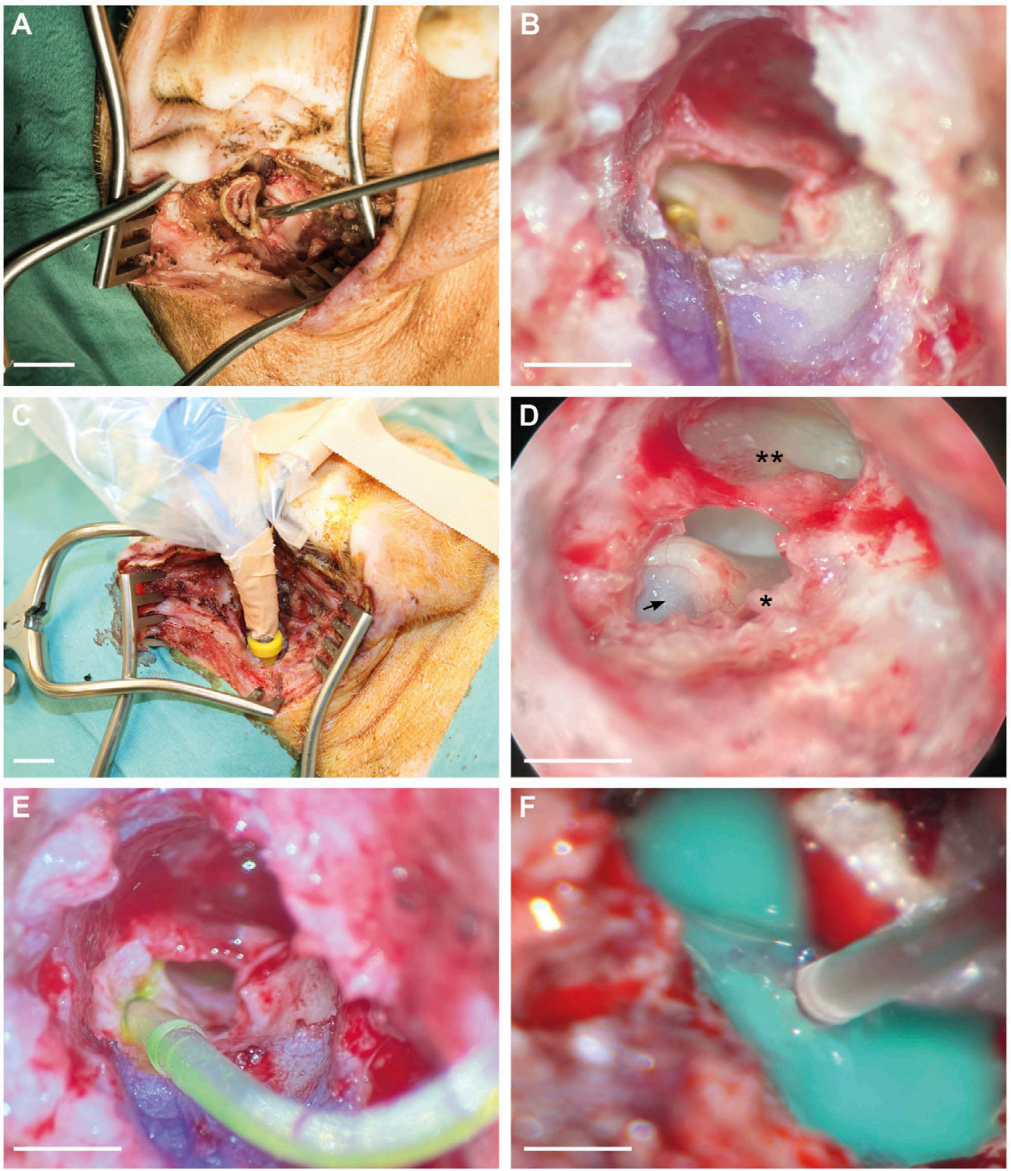
Surgical approach for CC insertion, gold wire fixation for ECochG recording, and PL sampling. **(A)** Endaural approach and view of the cartilaginous outer ear canal. **(B)** Gold wire electrode with contact to the RWM and fixation on the ear canal. **(C)** Intraoperative earphone insertion into the left ear canal of a piglet. **(D)** Microscopic view of the middle ear. The arrow indicates the entrance point of the CC through the round window. Asterisk (*) points out the short crus of the incus, and the double asterisk (**) refers to the tympanic membrane. **(E)** RWM insertion of the CC including FITC-d. **(F)** Apical PL sampling after the construction of a silicone cup. Scale bars: (A), (C) = 1 cm; (B), (D), (E) = 2 mm; (F) = 1 mm.

The surgical incision was performed in a circular shape underneath the tragus to further separate the cartilaginous part of the ear from the bony part (Figure 2A). The bony ear canal was drilled posteriorly and inferiorly to allow a more extensive view of the middle ear. The ear canal skin was cut with micro scissors just above the level of the anulus fibrosus to prevent any structural damage or perforation of the tympanic membrane. Bleeding was carefully cauterized, and the excessive ear canal skin was extracted, allowing an improved view of the ossicles and the RWM (Figure 2D). The small superior part of the tympanic membrane covers the approach to the round window. Therefore, this superior part was gently taken out, resulting in a view onto the RWM, ossicles, and tympanic membrane, as shown in Figure 2D. After fixing the gold wire for audiometry recordings, the earphone was inserted into the ear canal (Figure 2C), and hearing measurements were performed. At this point, stapes luxation and oval window membrane puncture was performed in the venting group only. Subsequently, the FITC-d-filled CC was inserted through the RWM, and three small pieces of the ear canal skin (each 0.5 mm in diameter) were placed around the CC onto the round window to prevent FITC-d backflow (Figure 2E). After the FITC-d injection, the CC was removed, and the RWM was sealed with ear canal skin and tissue glue. In case of stapes venting, the stapes was repositioned and the oval window membrane closed with ear canal skin. The above-mentioned hearing measurements were performed again shortly before PL sampling. If the drilled inferior bony ear canal did not allow the sight to the cochlear apex after removal of the tympanic membrane, excessive bone was further drilled. Malleus and incus were gently removed with micro forceps for better visualization of the bony capsule. Excessive mucosa was meticulously removed from the cochlear apex. The middle ear was then washed three times with sterile saline solution before the otic capsule of the cochlear apex was gently thinned with a cleaned 1 mm diamond burr. A small cup made of adhesive silicone glue was constructed, and the apex was gently perforated with a sterile needle. Finally, consecutive PL sampling was performed (Figure 2E).

### 3.2 Quantitative FITC-d analysis—total mean concentration comparison

**FIGURE 3:**
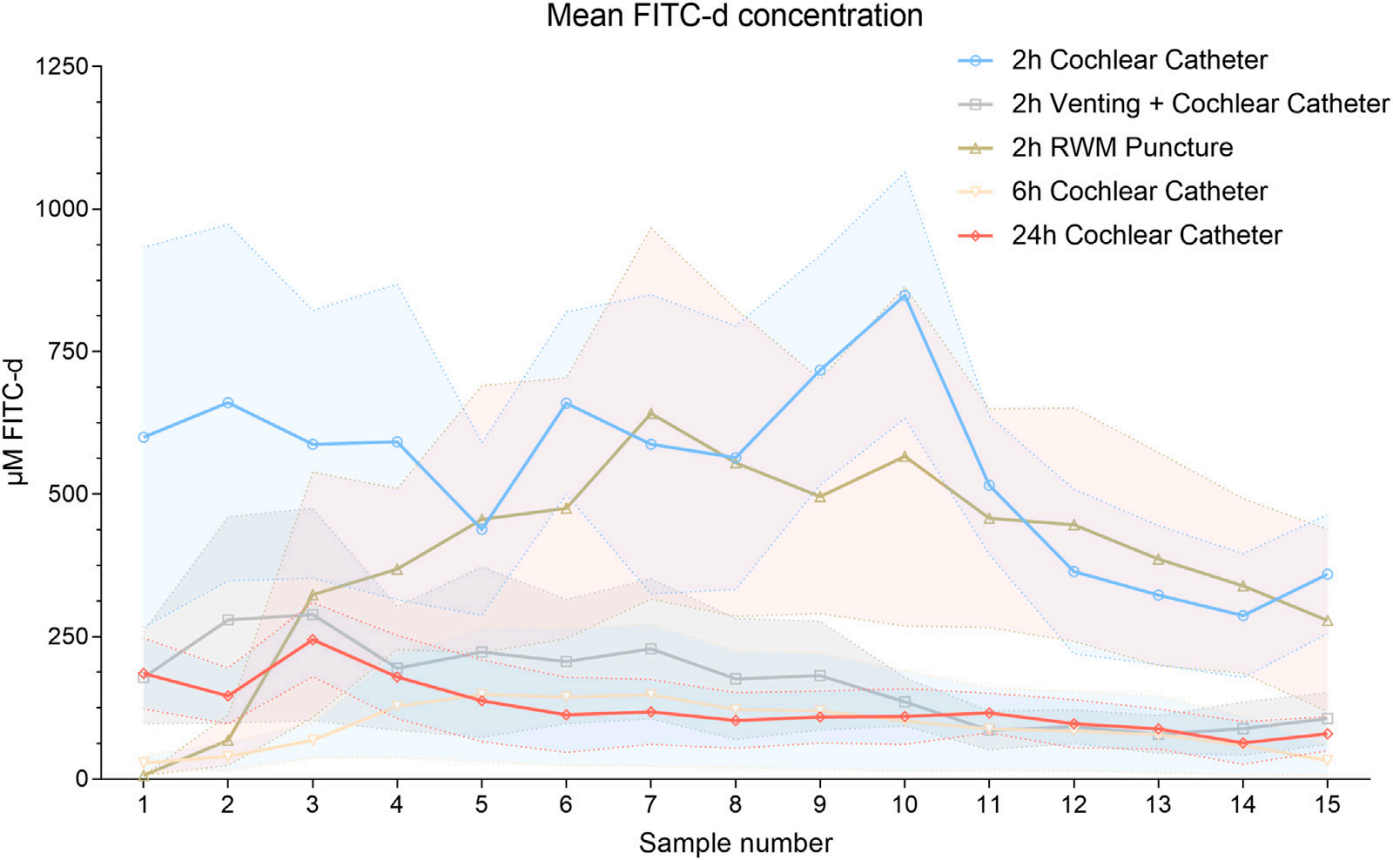
Mean FITC-d concentrations of each PL sample and group of all animals. Solid lines point out the mean values. The shaded error bars illustrate the SEM. Abbreviation: RWM, round window membrane.

Distribution of FITC-d in the inner ear was determined for each group with Figure 3 illustrating the compound’s varying concentrations in each piglet PL sample. The colored solid lines present each group’s mean and reveal the significant lower concentrations after longer observation periods, i.e., higher concentrations in the 2-h groups were detected compared to six- and 24-h observation times. Specifically, the mean FITC-d level of the 2 h CC group (540.18 μmol/l) was significantly higher than the FITC-d content in 6 h CC group (92.61 μmol/l, mean, *p* < 0.0001, 299.9—595.2 CI). Compared to the 24 h CC group (124.90 μmol/l, mean), the significant increase of FITC-d levels of the 2 h CC group remained (*p* < 0.0001, 266.7—561.9 CI). However, a FITC-d comparison of the 6 h CC group and the 24 h CC group resulted in no significant difference (*p* = 0.9720, −180.9–114.3 CI). Mean FITC-d concentrations in the 2 h CC group (540.18 μmol/l, see above) were significantly increased in comparison to the 2 h RWM group (290.93 μmol/l, mean, *p* = 0.0461, 1.6—296.9 CI). Both in the 2 h CC and the 2 h RWM group, significantly increased FITC-d concentrations were detected compared to the 2 h vent CC group (169.43 μmol/l, mean, 2 h CC, *p* < 0.0001, 223.1—518.4 CI; 2 h RWM, *p* = 0.0005, 73.9—369.1 CI). All mean values of each PL sample (1—15) were allocated to the respective groups and illustrated in Supplementary Figure S1.

### 3.3 Quantitative FITC-d analysis—comparison of apical, middle and basal PL samples

**FIGURE 4:**
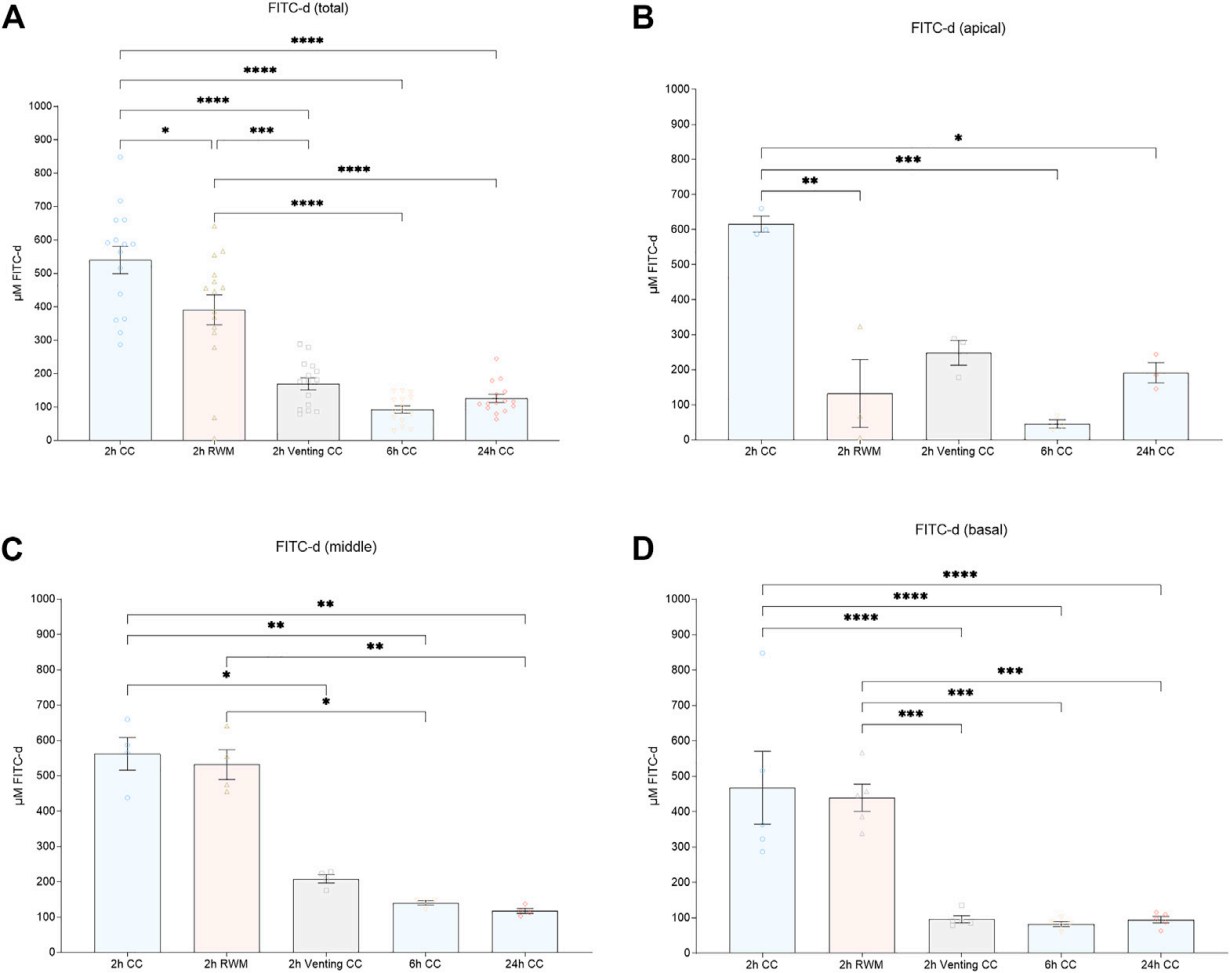
**(A)** Mean total FITC-d concentrations. Mean FITC-d values after division of PL samples in **(B)** apical (sample 1—3), **(C)** middle (sample 5—8), and **(D)** basal (sample 10—14) parts. Error bars indicate SEM. All asterisks represent significant differences between indicated groups (*p ≤ 0.05, **p ≤ 0.01, ***p ≤ 0.001, ****p ≤ 0.0001). Abbreviations: CC, cochlear catheter; RWM, round window membrane; Venting, venting group.

Dividing PL samples in putative apical (samples 1–3), middle (samples 5–8), and basal (samples 10–14) fractions according to their most probable location prior to the beginning of the sampling process resulted in significant differences between some groups, as shown in Figure 4. As outlined in the last paragraph, a tendency for more homogeneity of FITC-d concentrations with increasing observation times can be appreciated. Apical PL samples showed significantly higher FITC-d concentrations in the 2 h CC group (615.79 μmol/l, mean) *versus* the 2 h RWM group (132.71 μmol/l, mean, *p* = 0.0052, 108.6—857.5 CI), but not compared to the 2 h vent CC group (248.66 μmol/l, mean, *p* = 0.0573, −7.3—741.6 CI). In the other PL compartments (middle, samples 5—8, and basal, samples 10—14), there was no significant difference between the 2 h CC (middle 562.31 μmol/l, basal 467.53 μmol/l, mean) group and the 2 h RWM group (middle 531.92 μmol/l, mean, *p* = 0.9991, −307.8—368.6 CI; basal 439.07 μmol/l, *p* = 0.9961, −186.7—243.6 CI). Significantly lower concentrations in the 2 h vent CC group (middle 208.44 μmol/l, basal 96.01 μmol/l, mean) than in the 2 h CC group were detected (middle, *p* = 0.0357, 15.7—962.1 CI; basal, *p* < 0.0001, 156.4—586.7 CI). An illustration of all PL sample parts is presented in Figures 4B–D.

FITC-d levels of the apical PL sample fraction were significantly lower in the 6 h CC group (45.33 μmol/l, mean) than in the 2 h CC group (*p* = 0.0006, 196.0—944.9 CI). Accordingly, the FITC-d concentrations in the middle and basal PL sample fractions were significantly decreased in the 6 h CC group (middle, 140.51 μmol/l, mean, *p* = 0.0071, 83.59—760.0 CI; basal 82.23 μmol/l, *p* < 0.0001, 170.2—600.4 CI).

Compared to the 2 h CC group, apical PL sample fractions were significantly decreased in the 24 h CC group (191.93 μmol/l, mean, *p* = 0.0189, 49.4—798.3 CI). The significant decrease in the 24 h CC group in comparison to the 2 h CC group also remained in the middle (117.78 μmol/l, mean) and basal (94.81 μmol/l, mean) PL sample fractions (middle, *p* = 0.0039, 106.3—782.7 CI; basal, *p* < 0.0001, 157.6—587.8 CI).

### 3.4 Cochlear volume measurements–micro-CT scans

**FIGURE 5:**
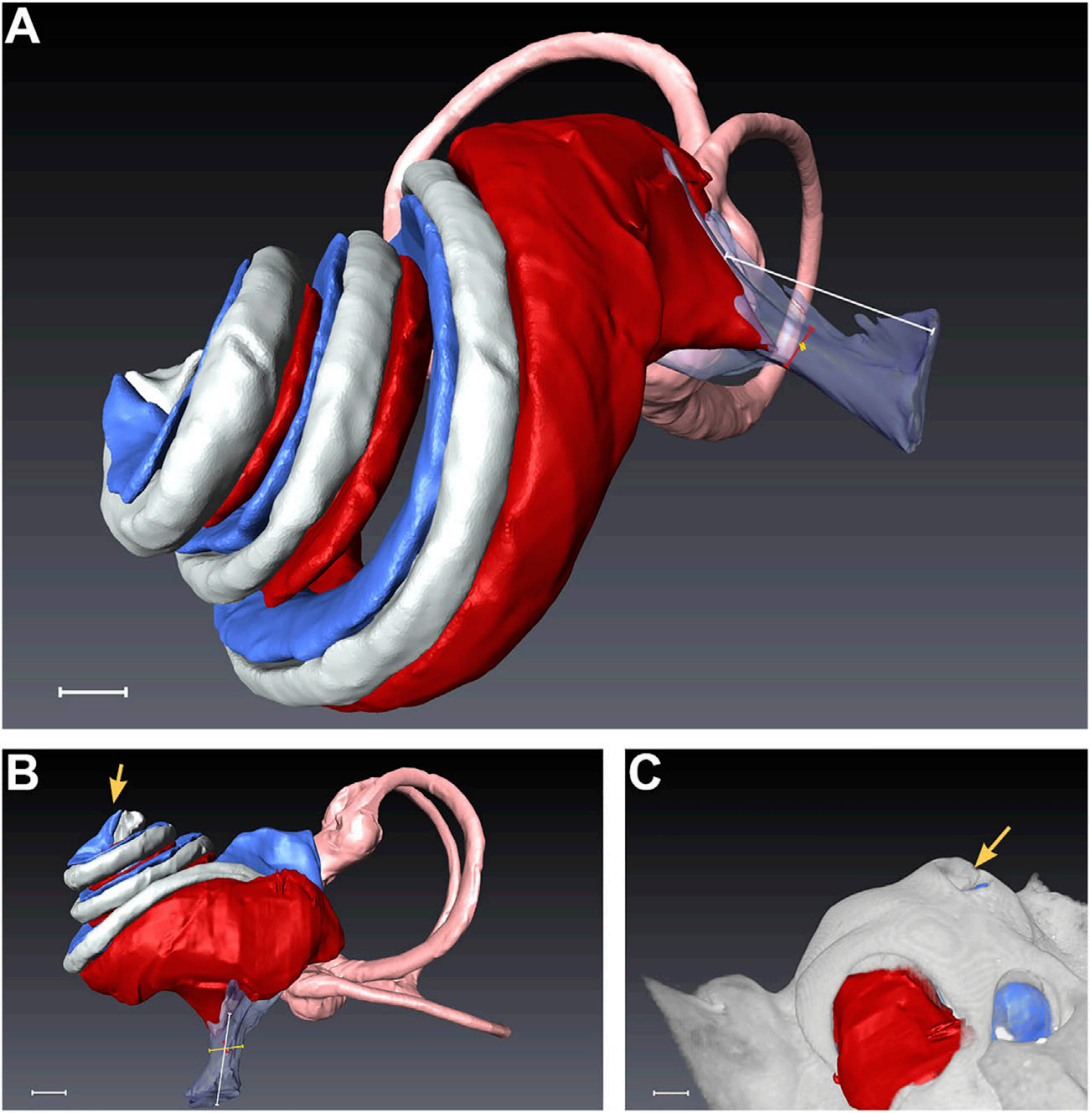
Segmentation of a randomly selected piglet cochlea after micro-CT scan. **(A)** depicts the overall side view, while **(B)** presents a direct view of the scala tympani. In **(C)**, the rendering of the bony capsule is matched with the segmented cochlear parts. The yellow arrows in **(B,C)** indicate the apical PL sampling hole, which was opened during the surgical intervention. Scale bar equals 1 mm. Red = scala tympani, pink = semicircular canals, transparent-magenta = cochlear aqueduct, gray = scala media, blue = scala vestibuli.

Datasets from randomly selected piglet cochleae were segmented for each above-mentioned cochlear compartment after successful PL sampling. Despite some variability in scalar volumes between the scanned inner ears, we could not detect any signs of pathologies or malformations in the analyzed specimens (see Supplementary Figure S2). All volume renderings of scanned cochleae revealed apical PL sampling holes, as pointed out in Figure 5. The summary in Table 1 shows the total inner ear volume of the segmented compartments ranging from 77.51 to 100.25 mm³ with an average of 84.55 ± 8.15 mm³ (mean ± SD, n = 5).

### 3.4 Objective hearing measurements—ECochG

**FIGURE 6:**
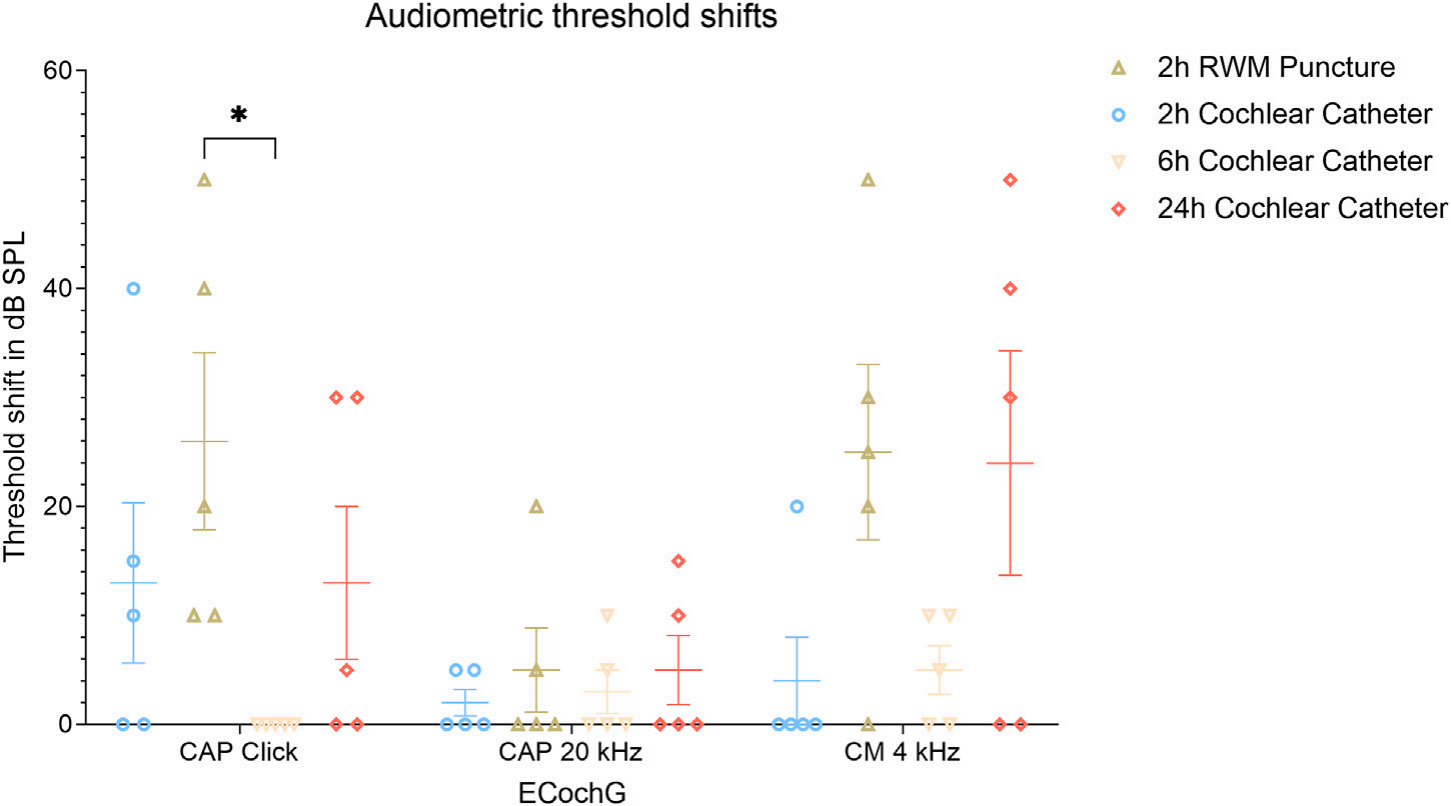
Audiometric threshold shifts for ECochG signals (click-CAP, tone burst 20 kHz and CM) divided into each group and including mean and SEM values. The asterisk outlines the significantly increased threshold shifts between the 6 h CC and the 2 h RWM groups with *p* ≤ 0.05. Abbreviations: ECochG, electrocochleography; RWM, round window membrane; SPL, sound pressure level.

There was no significant difference in threshold shifts of click-CAPs, 20 kHz CAPs, and CM between all groups with one exception when comparing the 2 h RWM group (26.0 ± 16.2 dB SPL, mean ± SD) with the 6 h CC group (no threshold shifts in click-CAPs determined), as the latter revealed significantly reduced threshold shifts (*p* = 0.0120, 4.5—47.5 CI). This was not observed in the threshold shift comparison of CMs (25 ± 16.1 dB SPL, 2 h RWM mean ± SD, *versus* 5.0 ± 4.5 dB SPL, 6 h CC mean ± SD, *p* = 0.0767, −1.5—41.5 CI), and of 20 kHz CAPs (5.0 ± 7.7 dB SPL, 2 h RWM mean ± SD, *versus* 3.0 ± 4.0 dB SPL, 6 h CC mean ± SD, *p* = 0.9946, −19.5—23.5 CI). Figure 6 depicts the threshold shifts of all groups and outlines the degree of hearing loss. In contrast to the 2 h RWM group, there was no significant difference in click-CAP threshold shifts between the 2 h CC group (13.0 ± 14.7 dB SPL, mean ± SD) and 6 h CC group (*p* = 0.3831, −8.5—34.5 CI). Further comparison of ECochG recordings of both groups resulted in no significant differences of CM (4.0 ± 8.0 dB SPL, 2 h CC mean ± SD, *p* = 0.9993, −22.5—20.5 CI) and 20 kHz CAP (2.0 ± 2.4 dB SPL, 2 h CC mean ± SD, *p* = 0.9993, -22-5—20.5) threshold shifts.

There were no significantly different threshold shifts of the respective ECochG recordings between the 2 h CC group and the 2 h RWM group (click-CAP 26.0 ± 16.2 dB SPL, 20 kHz CAP 5.0 ± 7.7 dB SPL, CM 25.0 ± 16.1 dB SPL, mean ± SD) as shown with *p* = 0.3831 (−34.5—8.5 CI) for click-CAP, *p* = 0.9823 (−24.5—18.5 CI) for 20 kHz CAP, and *p* = 0.0578 (−42.5—0.5 CI) for CM thresholds. No significant threshold shifts were detected between the 2 h CC and the 24 h CC group (click-CAP 13.0 ± 14.0 dB SPL, 20 kHz tone burst CAP 5.0 ± 6.3 dB SPL, CM 24.0 ± 20.6 dB SPL, mean ± SD) with *p* > 0.999 for click CAP (−21.5—21.5 CI), *p* = 0.9823 for 20 kHz CAP (−24.5—18.5 CI), and *p* = 0.0767 (−41.5—1.5 CI) for CM thresholds. A comparison of the 2 h RWM and the 24 h CC groups showed no difference in threshold shifts of CMs (*p* = 0.9993, −20.5—22.5 CI) and 20 kHz CAPs (*p* > 0.9999, −21.5—21.5 CI). An example of objective hearing measurements prior to FITC-d injection is shown in Supplementary Figure S3. Due to the traumatic stapes luxation in the venting group, no objective audiometric thresholds were recorded in this group.

## 4 Discussion

To the best of our knowledge, this is the first study that describes the intrascalar perilymphatic inner ear volumes of the pig in detail and furthermore directly addresses the inner ear distribution of the well-established marker compound FITC-d. Using segmented micro-CT data, we could show that the pig’s inner ear volume is larger than in other comparable animal models that have been evaluated so far and therefore appears to be closer to human dimensions. In another set of experiments, we demonstrate the feasibility of the atraumatic insertion (at the level of micro-CT images and electrophysiological measurements) of a CE-marked CC and drug administration through that device, highlighting the suitability of the chosen animal model for such studies. Finally, we were able to show that drug application using the CC results in higher apical FITC-d levels, which could have important clinical implications.

When performing pharmacokinetic studies in the inner ear, the choice of an adequate model is one of the most critical issues. Even though major cochlear structures are highly conserved across species (Burton et al., 2019), the size and configuration of the fluid-filled compartments vary significantly between different models (Glueckert et al., 2018). While the cochlea of rhesus macaques is 24 times larger than the murine inner ear, the human cochlear volume is about three times larger than that of macaques (Dai et al., 2017). The total inner ear volume of mice is around 2.5 µl (Dai et al., 2017), and it is considerably larger in guinea pigs with about 15.94 µl (Shinomori et al., 2001). In contrast, the segmented piglet inner ears in our study revealed a mean total inner ear volume of 84.55 µl, which is distinctly larger than the volume measured in rhesus macaques (59.4 µl) but still smaller than the human total inner ear volume, which covers 191.1 µl (Dai et al., 2017). Nevertheless, across the broad spectrum of species commonly used in translational hearing research, the piglet model appears to most closely resemble human cochlear dimensions.

Another essential aspect in translational inner ear therapy studies is the transferability of procedures and devices from the preclinical model into clinical applications. In this study, we therefore opted for a somewhat different approach. As there already is a clinically tested and CE-marked CC, we decided to use this device in the novel porcine model and we managed to establish the required surgical approach. Using this model, we were then able to perform the insertion of this catheter up to a depth of 12.5 mm without resistance. Relative atraumaticity of this procedure was confirmed by pre- and postoperative ABR measurements, which did not show increased hearing threshold shifts compared to the round window puncture group, as well as intact structures in the representative micro-CT scans (Supplementary Figure S4). In one CC-inserted group, the threshold shifts were even significantly reduced compared to the RWM injection. Considering threshold shift comparisons in other groups, we detected no significant changes between the CC groups and the RWM puncture group after ECochG signal recordings that tonotopically represent both basal and apical parts. However, these findings need to be interpreted with caution as we only gathered short-term data to determine acute changes in the hearing status. Longer observation times would be necessary to exclude potential differences in long-term hearing preservation rates.

Using direct intracochlear injections, we were able to show high perilymphatic FITC-d concentrations both after the application *via* the catheter and after RWM puncture. Total perilymphatic concentrations as well as apical levels (samples 1—3) of FITC-d were significantly higher after application *via* the CC. Thus, we were able to experimentally confirm the theoretically expected advantage of the CC *versus* standard injection through the RWM. Fluid oscillation across the CA permits substances to move from the CSF to the perilymphatic fluid space in the scala tympani and *vice versa*. As FITC-d is applied directly through the RWM in the control group, drainage *via* the relatively nearby CA (see Figure 6) might result from pressure changes in the scala tympani. This could explain an overall decrease of FITC-d content and might be the reason for lower mean concentrations of the samples, likely representing the perilymphatic space of the basal cochlear part. On the other hand, an implication of PL and CSF communication after the mechanical alteration of the RWM in guinea pigs was observed in a previous study (Plontke et al., 2016), in which dextran retention in the scala tympani was detected 40 min after application. Meanwhile, a CC insertion surpasses the CA and potentially leads to less drainage. It is assumed that the catheter insertion depth of 12.5 mm partially covers the basal cochlear turn in humans and piglets.

The achieved high apical drug levels are of particular interest in settings requiring treatment of the whole cochlea or precisely the cochlear apex, like gene therapy, or attempts to improve low frequency hearing preservation during cochlear implantation. Especially in the latter setting, a clinical application appears feasible because a surgical approach to the round window is created anyway, and the required additional trauma is therefore minimal. Furthermore, the risk/benefit ratio in these patients appears to be favorable due to the already existing profound hearing loss. Recently, first-in-human data regarding the CC have been published (Prenzler et al., 2018; Prenzler et al., 2020).

In an attempt to further increase apical FITC-d concentrations, we evaluated the effect of a vent hole in the oval window based on the surgical method in another large animal for adeno-associated vector application (Andres-Mateos et al., 2022). Due to anatomical restrictions in the porcine model, only a traumatic luxation of the stapes was feasible. Stapes venting resulted in our study in significantly decreased FITC-d levels, which is likely due to outflow through the vent hole during the injection procedure. We hypothesize that this led to an excessive loss of PL and hence to a reduction of total FITC-d concentrations in PL instead of the anticipated increase in apical PL concentrations of FITC-d. Therefore, the findings after performing a vent hole in other inner ear structures (e.g., semicircular canal) would be of interest in future studies. Due to clinical translational considerations, we pursued only injections without stapes venting for the 6h and 24 h experiments. Of note, FITC-d concentrations were significantly lower 6h and 24 h compared to 2 h after CC application. Two potential explanations for this observation include the loss of PL through the RWM (even though sealed meticulously) and/or the loss through diffusion/elimination. The greatest decline of dextran was observed in the basal and middle parts of the scala tympani in guinea pigs 2 hours after injection, which would support the rationale mentioned above (Salt et al., 2015). One potential elimination route could be the CA, which - comparable to the situation in humans - is located very near the porcine round window in the scala tympani. However, little is known about the patency of the structure in this animal model. Therefore, we cannot compute the elimination *via* this route, but we know that FITC-d in general is well retained in the PL space and has been used to study cochlear pharmacokinetics in other animal models (Salt and Hirose, 2018).

Randomly selected and rendered micro-CT scans (Supplementary Figure S1) and the microscopic view of the most apical cochlear part, which appeared slightly transparent, confirmed the apical bony capsule perforation for PL sampling in all animals. The choice of 15 consecutive 2 μl PL samplings results in a representation of 30 µl of inner ear fluid, which is thought to cover large parts of the perilymphatic space as confirmed by the volume measurements shown in Table 1 (e.g., scala tympani volume 30.11 ± 3.76 µl, mean ± SD). The detected FITC-d concentrations in our experiments serve as a basis for the investigation of future treatment options in large animal models, which can subsequently be translated to the clinical setting. The CC provides a unique opportunity for drug treatments that require higher apical drug concentrations. The applied CC insertion without stapes venting could be an optional drug delivery in patients with residual hearing during cochlear implantation surgery since an opening to the cochlea must be created during this procedure in all cases. In combination with the most appropriate electrode array design (Tarabichi et al., 2021), electro-acoustic stimulation may result in improved residual hearing outcomes in cochlear implant-treated patients due to efficient drug delivery to the low-frequency cochlear regions with the CC. This is especially important as intracochlear inflammation after cochlear implantation may be a major contributor to loss of acoustic hearing in those patients (Tejani et al., 2022).

Despite a thorough closure of the RWM in all animals, the significantly decreased contents of FITC-d in the 6 h- and 24 h-groups are worth mentioning. We observe the highest apical concentrations in the 2 h groups and a drastic decline in the 6 h- and 24 h-groups after single delivery *via* CC. In the last two groups, FITC-d was more evenly distributed across the inner ear compared to the shorter observation time. Hence, a relatively homogenous distribution of PL samples can be assumed with increasing time at least 6 h after local drug application *via* CC. Interestingly, the PL samples obtained after longer observation times were not significantly different regarding their FITC-d content. Manrique et al. confirmed in macaques that a continuous application (2 μl/h) of FITC-d with a 10 mM solution results in increased drug concentrations over time with an average value of 515.71 μmol/l. We observed a similar mean value in the corresponding first five samples (equal to 10 µl) with 575.43 μmol/l after a single 2.5 mM drug application *via* CC, which confirms the general comparability of both applied techniques despite their differences in methodology (Manrique-Huarte et al., 2021). The aforementioned homogeneity of FITC-d concentration in PL samples with increasing observation times was also observed in Manrique et al., where observation times up to 7 days were included. It is worth mentioning that a possible limitation of the PL sampling methodology might be caused due to varying sampling times. However, this factor had been observed in prior studies and seems to be dependent on the individual cochlear flow or the individual sampling hole diameter. Nevertheless, because of the comparability of Manrique’s study, the reproducibility of the method was confirmed.

Given the circumstance that the used CC is CE-marked, its use for intracochlear therapy may be indicated, e.g., in patients with a rupture of the RWM and subsequent sudden hearing loss, or as an additional treatment in patients who receive cochlear implants due to permanent functional hearing loss. According to current guidelines, idiopathic sudden sensorineural hearing loss can be treated with repeated intratympanic injections of steroids on consecutive days (Chandrasekhar et al., 2019). Another potential setting for intratympanic application of the same drugs was investigated in a study that carried out the intervention 1 day prior to cochlear implantation (Dahm et al., 2021). However, variance in drug diffusion and elimination after middle ear injection is accompanied by increased variability of intrascalar drug distribution as opposed to the direct injection into the PL (Salt et al., 2015). On the other hand, an application *via* CC is more invasive and therefore unlikely to be used repeatedly. We know from previous studies that a microcatheter fixation in the middle ears of patients may be accompanied by device dislocation, blood clots, and other adverse events (Plontke et al., 2006). This was also observed in combined strategies, such as the use of a controllable pump when coupled with a cochlear implant insertion, which was tested in macaques (Manrique-Huarte et al., 2021). Difficulty in fixation of the implant and catheter, device failures, and potential infections seem especially problematic in long-term intracochlear drug delivery, which subsequently does not appear clinically applicable today. In contrast, a single and short-term intracochlear drug application, e.g., with the help of previously described microneedles may lead to a reduced rate of infections and other adverse events (Aksit et al., 2021). However, the single controlled drug application *via* CC might be notably feasible due to a well-established application method with resemblance to cochlear implant surgery and significantly increased intracochlear drug concentrations.

## 5 Conclusion

Local drug delivery in patients with inner ear diseases remains an important topic that plays a major role in the field of pharmacokinetic hearing research as pathways and bioefficacy of pharmacological treatment need to be examined. This study describes the resulting FITC-d concentrations at multiple time points after intracochlear drug delivery *via* a clinically applicable CC in a large animal model with a high resemblance of dimensions to the human inner ear. We developed a novel surgical approach for local drug administration and apical PL sampling in piglets to translate results into the clinical setting, where optimal drug delivery is required for patients. With the emphasis on significantly higher FITC-d in the consecutively sampled inner ear fluids, especially those likely corresponding to the apical region of the inner ear, the drug concentrations are increased 2 hours after application and decrease significantly after 6 and 24 h with a more homogenous distribution after the latter two observation times. The reproducibility of the methodology and the presence of residual hearing after drug administration highlight the safety of CC insertion. Our study outlines promising clinically-relevant pharmacokinetic basics for inner ear treatment with otoprotective compounds. Its ramifications stand out as high drug concentrations in apical cochlear compartments are of relevance for the clinical setting. This circumstance is based on the fact that the spatial arrangement of sensory cells in those areas forward auditory signals to the brain that correspond to the human speech frequency.

## Data Availability

The original contributions presented in the study are included in the article/Supplementary Material, further inquiries can be directed to the corresponding author.

## Supplementary material

Supplementary Figure S1
**(A)** Individual and group FITC-d concentrations of all animals. **(B)** Mean FITC-d levels were divided into each group and PL sample (in µM). Colored and differently shaped symbols indicate group reference provided in Figure 1 of this manuscript. **(C)** illustrates the mean values of PL samples divided into apical (samples 1 – 3), middle (5 – 8), and basal (10 – 14). Abbreviations: CC, cochlear catheter; PL, perilymph; RWM, round window membrane; vent, venting.

Supplementary Figure S2
**(A–E)** Segmentation of piglet cochleae after micro-CT scan (direct view of the scala tympani). **(A’–E’)** Rendering of the bony capsule matched with the segmented cochlear parts. The yellow arrows indicate the apical PL sampling hole, which was opened during the surgical intervention. Scale bars equal 1 mm with the exception for B’ (2 mm) and for A’ (2.5 mm). Red, scala tympani; pink, semicircular canals; transparent-magenta, cochlear aqueduct; gray, scala media; blue, scala vestibuli.

Supplementary Figure S3 Randomly selected objective hearing measurements prior to FITC-d injection. With decreasing intensity in dB SPL, the **(A)** click-CAP and **(B)** CM recordings are presented. Abbreviations: CAP, compound action potential; CM, cochlear microphonic.

Supplementary Figure S4 Representative mid-modiolar section of a porcine inner ear in which perilymph sampling and further micro-CT scanning was performed. Cochlear basal, apical and modiolar regions are labeled. The enlarged picture of the basal region on the right highlights the organ of Corti in red, the lateral wall in orange and Reissner’s membrane in blue. Scale bars equal 0.5 mm. Abbreviations: ST, Scala tympani; SM, Scala media; SV, Scala vestibuli.

## Abbreviations

BW, Body weight; CA, Cochlear aqueduct; CC, Cochlear catheter; CAP, Compound action potential; CI, 95% confidence interval; click-ABR, Click evoked auditory brainstem response; CM, Cochlear microphonic potential; CSF, Cerebrospinal fluid; ECochG, Electrocochleography; FITC-d, Fluorescein isothiocyanate-dextran; PL, Perilymph; RWM, Round window membrane; SD, Standard deviation; SEM, Standard error of the mean; SPL, Sound pressure level; vent, Venting.
