# Supplementary material for: Investigation of inner ear drug delivery with a cochlear catheter in piglets as a representative model for human cochlear pharmacokinetics
Source: Front Pharmacol. 2023 Mar 9;14:1062379. doi: 10.3389/fphar.2023.1062379 (PMC10034346; doi:10.3389/fphar.2023.1062379)
Supplement: Supplementary file 1 [file DataSheet1.pdf]

Investigation of Inner Ear Drug Delivery with a Cochlear Catheter in Piglets as a Representative Model for Human Cochlear Pharmacokinetics

Erdem Yildiz[1,2], Anselm J. Gadenstaetter[1,2], Matthias Gerlitz[1,2], Lukas D. Landegger[1,2], Rudolfs Liepins[2], Michael Nieratschker[1,2], Rudolf Glueckert[3], Hinrich Staecker[4], Clemens Honeder[1,2*], Christoph Arnoldner[1,2*†]

[1] Christian Doppler Laboratory for Inner Ear Research, Department of Otorhinolaryngology, Vienna General Hospital, Medical University of Vienna, Vienna, Austria

[2] Department of Otorhinolaryngology – Head and Neck Surgery, Vienna General Hospital, Medical University of Vienna, Vienna, Austria

[3] Department of Otorhinolaryngology, Medical University of Innsbruck, Innsbruck, Austria

[4] Department of Otolaryngology – Head and Neck Surgery, University of Kansas School of Medicine, Kansas City, USA

*These authors contributed equally.

†Correspondence:
Assoc. Prof. Priv.-Doz. Dr. Christoph Arnoldner, MBA
christoph.arnoldner@meduniwien.ac.at
Phone: +43 1 40400 560 70
Waehringer Guertel 18-20
1090 Vienna
Austria

Supplementary Materials

Supplementary Figure 1

A

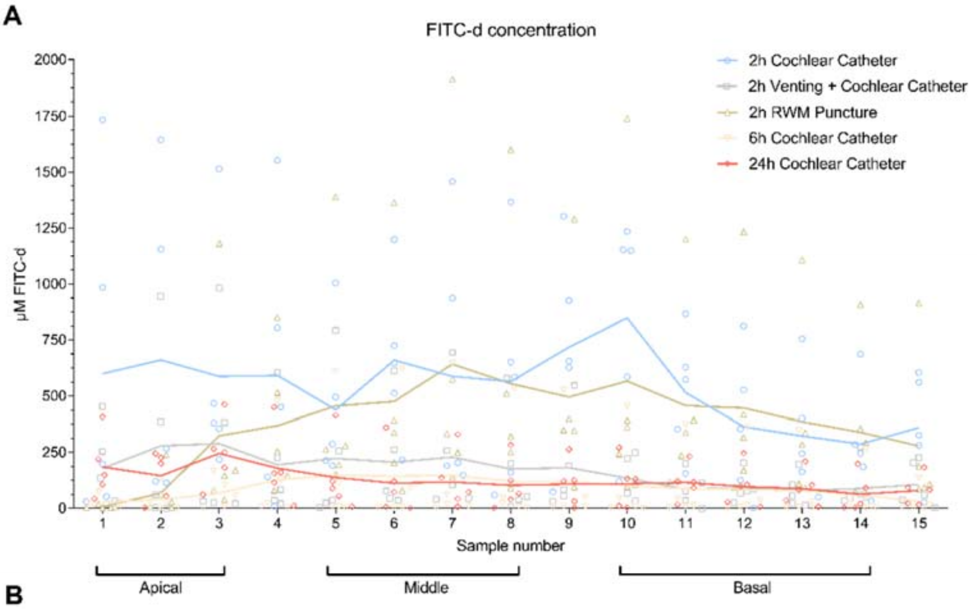

B

	Mean FITC-d (in µM) for each PL sample and group														
	1	2	3	4	5	6	7	8	9	10	11	12	13	14	15
	599.61	660.47	587.30	591.57	438.18	659.59	587.26	564.18	717.24	848.24	515.55	364.01	322.84	286.99	359.68
	178.28	279.10	288.59	194.11	223.00	206.34	228.44	176.00	181.42	135.29	85.48	91.97	78.69	88.61	106.09
	6.48	68.32	323.35	368.69	455.96	475.35	641.56	554.79	495.67	566.46	457.50	446.28	386.09	339.02	278.44
	28.28	39.52	68.19	127.81	148.12	144.07	147.68	122.18	119.66	101.71	88.07	84.26	78.08	59.04	32.44
	185.18	145.89	244.71	179.13	137.46	112.56	118.10	102.97	108.78	109.80	115.91	97.14	88.05	63.17	79.65

C

	Apical		Middle		Basal
2h CC	615.79		562.31		467.53
2h vent CC	248.66	p=0.0052	208.44		96.01
2h RWM	132.71		531.92		439.07
6h CC	45.33		140.51		82.23
24h CC	191.93		117.78		94.81

Investigation of Inner Ear Drug Delivery with a Cochlear Catheter in Piglets as a Representative Model for Human Cochlear Pharmacokinetics

Supplementary Figure 1: (A) Individual and group FITC-d concentrations of all animals. **(B)** Mean FITC-d levels were divided into each group and PL sample (in µM). Colored and differently shaped symbols indicate group reference provided in Figure 1 of this manuscript. **(C)** illustrates the mean values of PL samples divided into apical (samples 1 – 3), middle (5 – 8), and basal (10 – 14). Abbreviations: CC = cochlear catheter, PL = perilymph, RWM = round window membrane, vent = venting.

Supplementary Figure 2

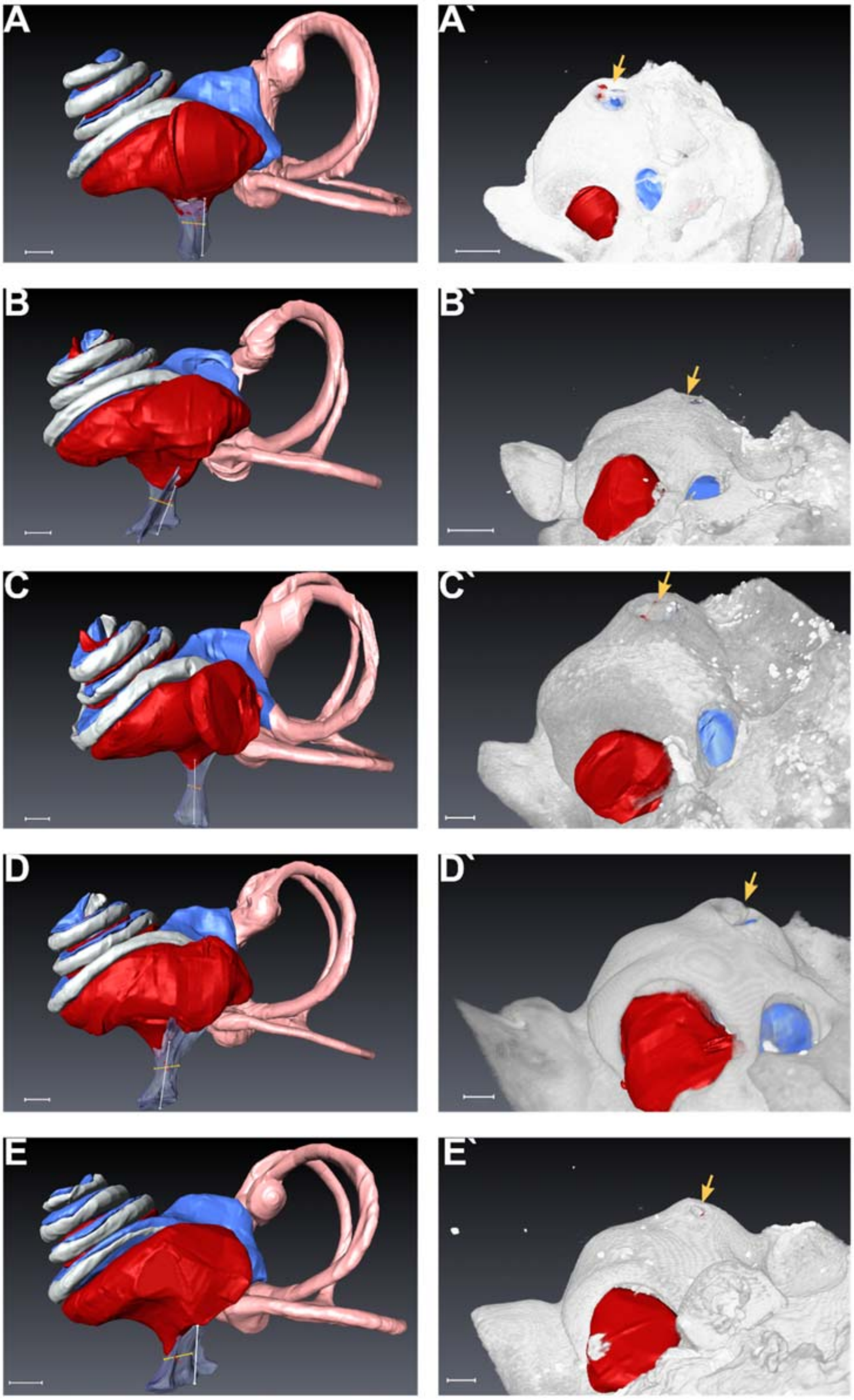

Supplementary Figure 2: (A-E) Segmentation of piglet cochleae after micro-CT scan (direct view of the scala tympani). **(A'-E')** Rendering of the bony capsule matched with the segmented cochlear parts.

The yellow arrows indicate the apical PL sampling hole, which was opened during the surgical intervention. Scale bars equal 1 mm with the exception for B’ (2 mm) and for A’ (2.5 mm). Red = scala tympani, pink = semicircular canals, transparent-magenta = cochlear aqueduct, gray = scala media, blue = scala vestibuli.

Supplementary Figure 3

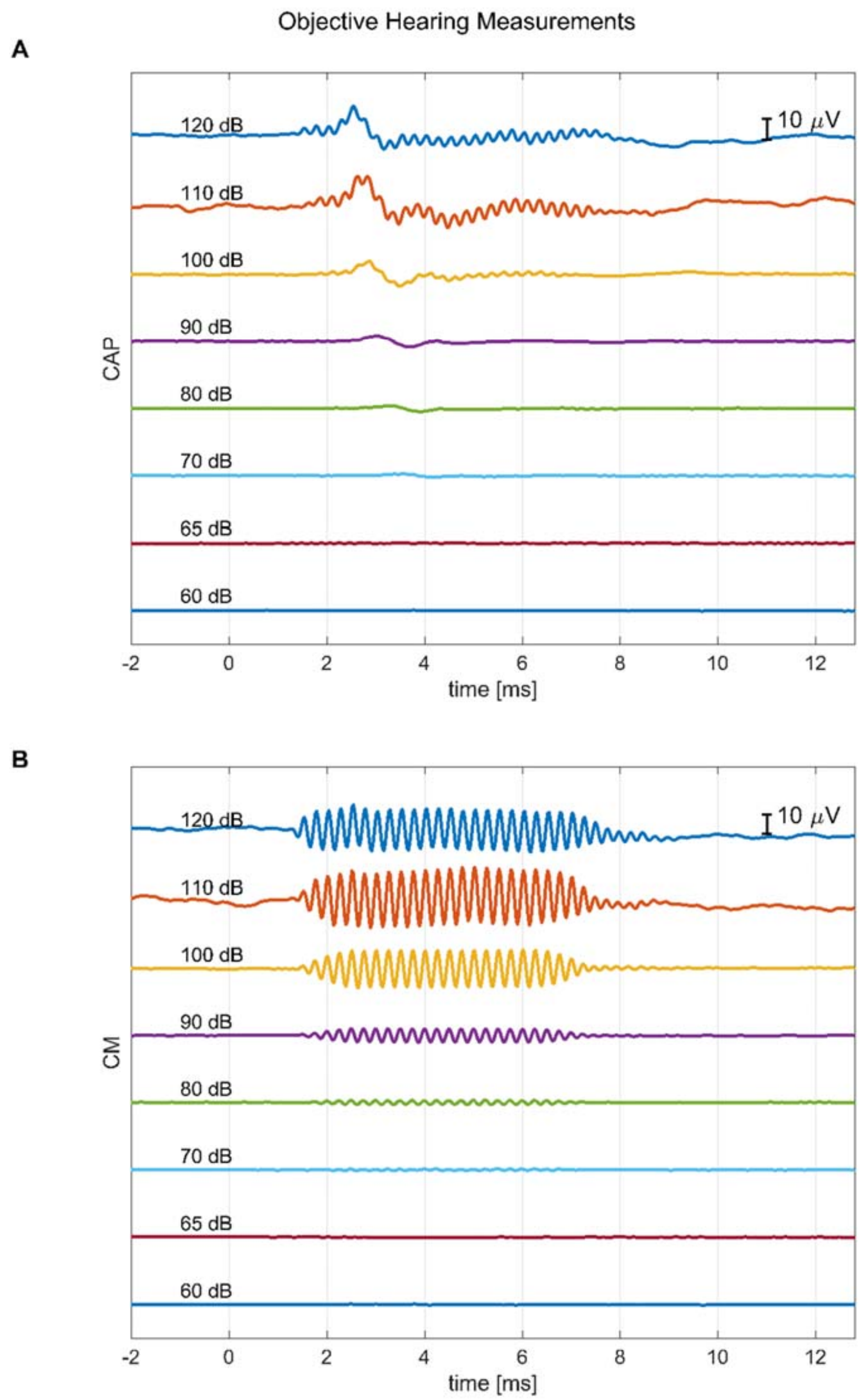

Supplementary Figure 3: Randomly selected objective hearing measurements prior to FITC-d injection. With decreasing intensity in dB SPL, the **(A)** click-CAP and **(B)** CM recordings are presented. Abbreviations: CAP = compound action potential, CM = cochlear microphonic.

Investigation of Inner Ear Drug Delivery with a Cochlear Catheter in Piglets as a Representative Model for Human Cochlear Pharmacokinetics

Supplementary Figure 4

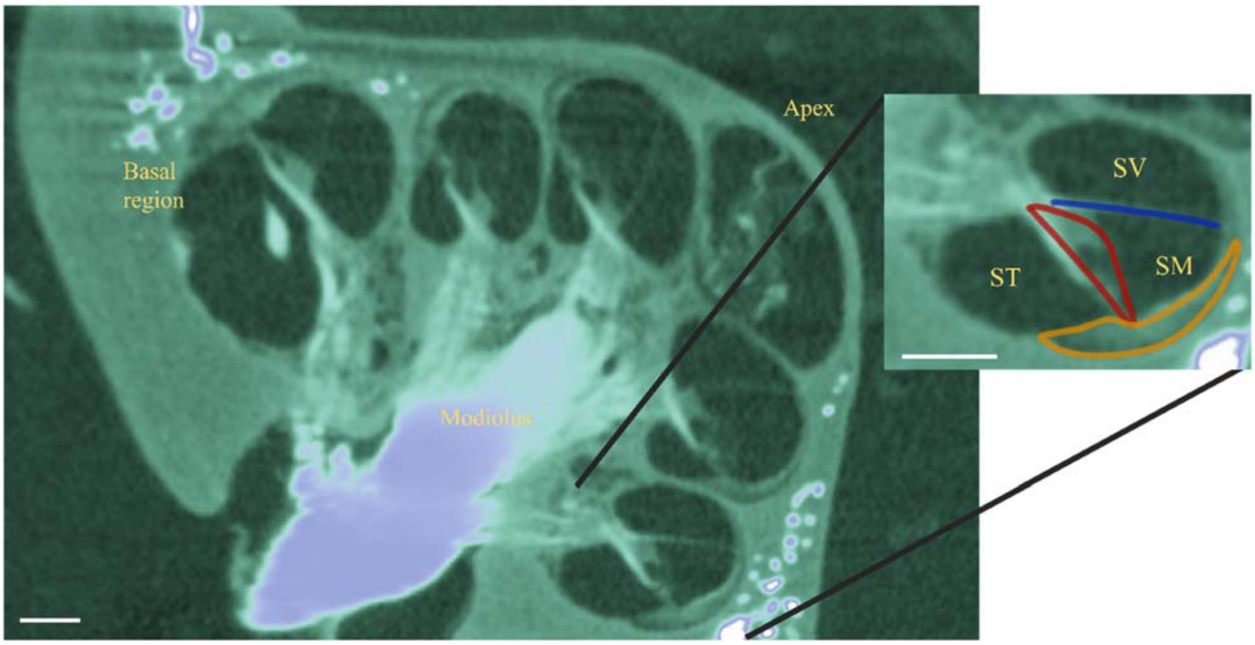

Supplementary Figure 4: Representative mid-modiolar section of a porcine inner ear in which perilymph sampling and further micro-CT scanning was performed. Cochlear basal, apical and modiolar regions are labeled. The enlarged picture of the basal region on the right highlights the organ of Corti in red, the lateral wall in orange and Reissner's membrane in blue. Scale bars equal 0.5 mm. Abbreviations: ST = Scala tympani, SM = Scala media, SV = Scala vestibuli.
